# No Forest Left Behind

**DOI:** 10.1371/journal.pbio.0050216

**Published:** 2007-08-14

**Authors:** Gustavo A. B da Fonseca, Carlos Manuel Rodriguez, Guy Midgley, Jonah Busch, Lee Hannah, Russell A Mittermeier

## Abstract

Mitigating climate change by reducing deforestation should involve incentives for countries that currently have high forest cover and low deforestation rates.

New research indicates that slowing tropical deforestation may play a much larger role in mitigating climate change than previously believed [1,2]. Carbon emissions from tropical deforestation are expected to increase atmospheric CO_2_ concentration by between 29 and 129 ppm within 100 years, much more than previously estimated [3]. The parties to the United Nations Framework Convention on Climate Change are considering policy approaches and incentives for reducing emissions from deforestation (RED) in developing countries [4–6] that are timely, in light of these recent research findings. The leading proposals would enable trading of carbon saved by reducing tropical deforestation, just as carbon is currently traded from reducing industrial emissions. The state of these discussions suggests that a key group of countries are at risk of being omitted from a new framework—those with high forest cover and low rates of deforestation (HFLD).

Developing countries can be classified into four categories defined by two axes: remaining forest cover and deforestation rate (Figure 1). The HFLD countries in Quadrant IV harbor 18% of tropical forest carbon. Since current proposals would award carbon credits to countries based on their reductions of emissions from a recent historical reference rate [4], HFLD countries could be left with little potential for RED credits. Nor would they have the potential for reforestation credits under the Kyoto Protocol's Clean Development Mechanism that the countries in Quadrant II have. Without the opportunity to sell carbon credits, HFLD countries would be deprived of a major incentive to maintain low deforestation rates. Since drivers of deforestation are mobile, deforestation reduced elsewhere could shift to HFLD countries, constituting a significant setback to stabilizing global concentrations of greenhouse gases at the lowest possible levels.

**Figure 1:**
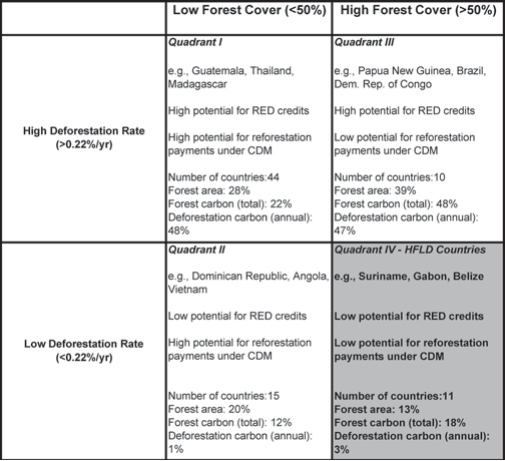
Forests and Carbon in 80 Tropical Countries The term HFLD is applied to those countries that had forest cover greater than 50% in 2005 and average annual deforestation rates lower than the global average of 0.22% during the reference emission period of 1990–2000 (Quadrant IV). Values for forest cover, forest area, deforestation rate, and above ground biomass in forests are from [10]. “Forest area” denotes a quadrant's share of the 80 countries' forest area. “Forest carbon” denotes a quadrant's share of the 80 countries' forest carbon stock (above ground biomass). “Deforestation carbon” denotes a quadrant's share of the 80 countries' annual carbon emissions from deforestation. CDM, Clean Development Mechanism; RED, reducing emissions from deforestation.

An effective RED carbon regime should not allow leaks of deforestation to new regions, but should reduce net global emissions by encouraging comprehensive changes in international behavior. Some analysts have proposed adopting a reference emission rate indexed to the global deforestation rate for countries with little or no historic deforestation [7,8]. This would effectively award HFLD countries with “preventive credits” that these countries would stand to forfeit if they were to increase their deforestation rate. Preventive credits would provide a significant entry barrier to new forest exploitation or policies that promote or allow deforestation.

HFLD countries would receive a significant incentive to maintain low rates of deforestation from any reference emission rate indexed to the global average. At US$10/ton CO_2_, using one-third of the global average deforestation rate as the reference emission rate for HFLD countries, preventive credits would be worth US$365 million annually to seven countries. Using one-half of the global average deforestation rate as the reference rate would more than double the qualifying forest area, and would increase credit value to US$630 million annually to ten countries (Table 1). Using the global average deforestation rate as the reference rate would increase credit value to US$1.8 billion annually to 11 countries.

**Table 1:**
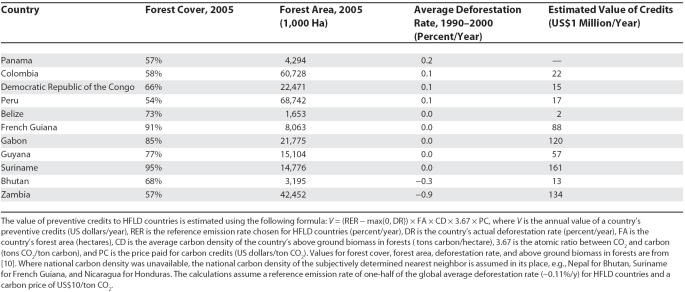
Estimated Annual Value of Preventive Credits

Despite the advantages of crediting for HFLD countries, some practical concerns remain. Introducing an additional source of carbon credits could lower the price of carbon, weakening the incentive to reduce deforestation in countries where rates are high. However, preventive credits should be evaluated in light of their net effect in reducing global CO2 emissions. The volume of preventive credits necessary to create an advance incentive against deforestation in HFLD countries would be 10–49 million tons of carbon annually, depending on which reference rate is selected. This is equivalent to just 1.3%–6.5% of developing countries' emissions from deforestation. The greater the global demand for carbon credits, the less impact this increase in supply would have on carbon price. In return, preventive credits would extend substantial protection to nearly one-fifth of tropical forest carbon.

Finally, countries like Brazil, Indonesia, and the Democratic Republic of the Congo are so large that they have regions in multiple quadrants. The Brazilian Amazon has attributes similar to the countries in Quadrant IV, and RED credits are being negotiated for the region [9]. While the quadrant approach helps identify technical gaps and policy options, in practice international responses must be tailored to individual country realities.

Preventive credits are an important part of a realistic approach to quickly minimize carbon releases from loss of some of the world's most biologically important forests. Globally indexed reference emission rates for HFLD countries should be part of any international framework for reducing global carbon emissions from deforestation.
